# *Lactobacillus delbrueckii* might lower serum triglyceride levels *via* colonic microbiota modulation and SCFA-mediated fat metabolism in parenteral tissues of growing-finishing pigs

**DOI:** 10.3389/fvets.2022.982349

**Published:** 2022-09-29

**Authors:** Gaifeng Hou, Jie Yin, Liangkai Wei, Rui Li, Wei Peng, Yong Yuan, Xingguo Huang, Yulong Yin

**Affiliations:** ^1^Key Laboratory of Agro-Ecological Processes in Subtropical Region, Hunan Provincial Key Laboratory of Animal Nutritional Physiology and Metabolic Process, National Engineering Laboratory for Poultry Breeding Pollution Control and Resource Technology, Hunan Research Center of Livestock and Poultry Sciences, South Central Experimental Station of Animal Nutrition and Feed Science in the Ministry of Agriculture, Institute of Subtropical Agriculture, Chinese Academy of Sciences, Changsha, China; ^2^College of Animal Science and Technology, Hunan Co-Innovation Center of Animal Production Safety, Hunan Agricultural University, Changsha, China

**Keywords:** *Lactobacillus delbrueckii*, lipid metabolism, SCFAs, colonic microbiota, growing-finishing pigs

## Abstract

Gut microbiota and its metabolites play a key role in host metabolism. Our previous study found supplemental *Lactobacillus delbrueckii* affected lipid metabolism of pigs, however, the underlying mechanism is unclear. In this study, we investigated the effects of *L. delbrueckii* on colonic bacteria composition and its metabolites, serum lipids and hormone levels, fat metabolism related enzyme activity and gene expression in various tissues of growing-finishing pigs. Twelve pigs were randomly distributed into two groups (*n* = 6), and pigs in each group were fed diets with (Con + LD) or without (Con) 0.1 % *L. delbrueckii* for 28 days. Results exhibited the deceased triglyceride (TG) levels and elevated free fatty acid (FFA) contents in serum and increased concentrations of butyric acid in colonic digesta after *L. delbrueckii* supplementation. Dietary *L. delbrueckii* increased abundance of *Lactobacillus* and *Butyrivibri* and tended to increase abundance of *Akkermansia and Megasphaera* in colonic digesta. *L. delbrueckii* consumption up-regulated glucagon-like peptide1 (*GLP-1*), monocarboxylate transporter1 (*MTC1)* and sodium-dependent monocarboxylate transporter1 (*SMCT1*) expression in colonic tissue. Administration of *L. delbrueckii* tended to increase lipoprotein lipase (LPL) activity, up-regulated *CPT-1*, angiopoietin-like protein 4 (*Angpt14*), *LPL* and triglyceride hydrolase (*TGH*) expression and down-regulated fatty acid synthetase (*FAS*), G protein-coupled receptor 41(*GPR41*) and *GPR43* expression in the liver. *L. delbrueckii* addition increased adenosine monophosphate activated protein kinase (*AMPK*) expression in *longissimus dorsi*, upregulated *LPL, CPT-1, Angptl4* and cluster of differentiation 36 (*CD36*) expression in subcutaneous fat, and enhanced *LPL, CPT-1, TGH*, adipocyte determination and differentiation-dependent factor 1 (*ADD1*) and hormone sensitive lipase (*HSL*) expression in leaf lard. These findings suggested that *L. delbrueckii* might enhance lipolysis and fatty acid β-oxidation to lower serum TG levels via colonic microbiota modulation and short chain fatty acids-mediated lipid metabolism of growing-finishing pigs.

## Introduction

In China, an increasing proportion of meat products appears in human diets as upturn living standards and rapid economic development in recent decades (1). Pork is a good source of protein and its global production and consumption is about 103.8 million tons in 2021 (2). China is the largest producer and consumer of pork in the world, nearly accounting for 50% of global total, and the annual pork consumption of individual residents is about 40 kg (3, 4). Pork quality directly correlates with human health. Long-term excessive intake of pork and its products is often associated with overconsumption of energy and fat, resulting in excess weight, obesity and an increased risk of some types of chronic disease, such as hyperlipidemia, cardiovascular disease (CVD) and type 2 diabetes, usually characterized by high levels of blood total cholesterol (TC) and TG (5, 6). Adipose tissue deposition is an important factor influencing pork quality (7), and knowledge on the fat metabolism and the mechanisms of fat deposition in pigs is very important for us to develop nutritional manipulations of pork quality.

In recent years, numerous researches on germ-free animals, fecal microbiota transplantation (FMT), probiotics treatment and microbiome analysis have widely confirmed that alterations of microbial ecology contribute to host fat metabolism (8–10). The body fat rate is associated with the intestinal Bacteroidetes and Firmicutes abundance, and lower abundance of Bacteroidetes and higher Firmicutes abundance were found in obese pigs relative to the lean pigs (11). Differences in fat deposition between lard type and lean pigs are closely correlate their gut microbiota, and obesity-associated phenotypes from donors to recipients can be transferred *via* FMT (12). Additionally, metabolites of gut microbes, such as short chain fatty acids (SCFAs) not only is of importance in gut health as signaling molecules, but also might enter the systemic circulation and directly affect the metabolism or function of peripheral tissues (13). SCFAs is considered as a bridge linking gut microbiota and parenteral tissues (13), which exhibit a vital role in fat accumulation *via* affecting lipid metabolism, energy metabolism and appetite (14). In recent years, an increasing attention are paid on intervention studies of probiotics influencing lipid metabolism *via* manipulation of gut microbiota and metabolites. Accumulating evidences have confirmed that *Lactobacillus* and its related products can effectively regulate lipid metabolism *via* affecting intestinal nutrition digestion and absorption, gut microbiota and metabolites, tissue gene expression and enzyme activity (15, 16). *Lactobacillus delbrueckii* as a *lactobacillus* strain is widely used in the dairy industry for yogurt and cheese (17, 18). Our laboratory have identified and preserved a strain of *L. delbrueckii* and performed a series of researches to evaluate its role on swine production since 2009. Last 5 years, our studies demonstrated that dietary *L. delbrueckii* can lower serum TC and TG levels, reduce fat deposition and improve meat quality of pigs (5, 19–21), but its underling mechanism is still ambiguity.

Given that growing-finishing pigs have a strong capacity of fat deposition and can be a good model for investigation about *Lactobacillus* regulating lipid metabolism. We hypothesized inclusion of *L. delbrueckii* would alter colonic bacterial composition and structure, thereby affecting bacterial fermentation and their metabolites (SCFA profile), and regulating lipid metabolism of pigs. Therefore, we investigated the effects of *L. delbrueckii* on colonic bacteria composition and its metabolites, serum lipids and hormone levels, lipid metabolism related enzyme activity and gene expression in different tissues of growing-finishing pigs.

## Materials and methods

All animal care, handling, and surgical techniques followed protocols approved by the Animal Care and Use Committee of the Institute of Subtropical Agriculture, Chinese Academy of Sciences under the permit number IACUC#201302 (Changsha, China). *Lactobacillus delbrueckii* was provided by the microbiology functional laboratory of the College of Animal Science and Technology in the Hunan Agricultural University (Changsha, China). The strain was activated and sent to the PERFLY-BIO (Changsha, China) for large-scale production, and the viable count of final products reached 5 × 10^11^ CFU/g.

### Animals, diets and experimental design

Twelve crossbred barrows (Landrace × Yorkshire) with an average initial body weight of (38.70 ± 5.33) kg were randomly assigned to 2 groups with 6 pigs, and all pigs were individually housed in the metabolism crates (1.4 × 0.7 × 0.6m) equipped with a feeder, a nipple drinker and a fully slatted plastic floor. Pigs were fed a basal diets supplemented with either 0 (Con) or 0.1% *L. delbrueckii* (5 × 10^10^ CFU/g, Con + LD) for 28 days. The basal diets (Table 1) were formulated to satisfy or exceed the nutritional requirement recommendation for 50- to 75-kg pigs (22). All pigs were fed their respective diets twice each day (8:00 a.m. and 15:00 p.m.) and had free access to water. All pigs were individually weighed at the beginning and end of the experiment, and the daily feed consumption per pig was recorded during the experimental period.

**Table 1:** Ingredients composition and nutritional levels of basal diets (air-dry basis, %).

**Ingredients**	**Contents**
Corn	66.76
Wheat middling	4.00
Wheat bran	6.00
Soybean meal (43% crude protein)	18.00
Soybean oil	1.00
L-lysine	0.24
Premix^a^	4.00
Total	100.00
Calculated nutritional levels
Digestible energy (DE, kcal/kg)	3,413.79
Crude protein	14.82
Standardized ileal digestible lysine (SID Lys)	0.85
Calcium	0.60
Total phosphorus	0.55

^a^The premix provided the following per kg of diet: VA 2,512 IU, VD3 1,200 IU, VE 34 IU, VK3 1.5 mg, VB12 17.6 μg, lactoflavin 2.5 mg, pantothenic acid 6.8 mg, niacin 20.3 mg, choline chloride 351 mg, Mn 10 mg, Fe 50 mg, Zn 50 mg, Cu 20 mg, I 0.3 mg, Se 0.3 mg.

### Sample collection and preparation

On day 29, the jugular vein blood samples were collected from the fasting pigs before slaughter using electrical stunning. Serum was obtained, aliquoted, and stored at −20°C for lipid, hormone and SCFAs analysis. Digesta (in colon) and tissues (in colon, liver, *longissimus dorsi*, subcutaneous fat and leaf lard) were quickly removed, snap frozen in the liquid nitrogen, and stored at −80°C for microbiota composition, SCFAs, enzyme activity and gene mRNA expression measurements.

### Measurement of serum lipids and hormone levels

Serum concentrations of triglyceride (TG), glucose (GLU) and free fatty acid (FFA) were detected using the BS 200 automatic blood biochemical analyzer (Mindray, Shenzhen, China) with corresponding kits. Serum levels of insulin (INS), leptin (LEP), peptide tyrosine tyrosine (PYY) and glucagon-like peptide-1 (GLP-1) were determined according to the instruction of corresponding commercial ELISA Kits (Jiangsu Yutong Biological Technology Co., Ltd., Jiangsu, China).

### SCFAs profiles analysis in serum and colonic digesta

Measurement of acetic acid, propionic acid and butyric acid contents in serum and colonic digesta were performed using an Agilent 7890A gas chromatographer (Agilent Technologies Inc., Palo Alto, CA, USA) equipped with an HP-FFAP elastic quartz capillary vessel column (30 m × 0.25 mm × 0.25 μm) according to the method detailly described in our previous studies (23, 24).

### Determination of hepatic enzyme activity related to lipid metabolism

Hepatic total protein contents (g protein/L) were quantified using a BCA protein assay reagent kit (Nanjing Jiancheng Bioengineering Institute, Nanjing, China), and the concentrations of hepatic fatty acid synthetase (FAS, U/g.prot), hormone sensitive lipase (HSL, U/g.prot), lipoprotein lipase (LPL, U/g.prot) and adipose triglyceride lipase (ATGL, IU/g.prot) were measured using corresponding commercial ELISA Kits (Jiangsu Yutong Biological Technology Co., Ltd., Jiangsu).

### Colonic bacterial composition and structure

Colonic microbiota composition and structure were indentified according to our previous description (5). Briefly, total DNA was extracted and purified from colonic digesta samples (*n* = 6 pigs/group) using a TIANamp Stool DNA kit (Tiangen Biotech (Beijing) Co., Ltd, China). DNA quality and quantity were evaluated by gel electrophoresis and a NanoDrop ND-1000 spectrophotometer (Thermo Fisher Scientific, USA), respectively. However, one low-quality DNA sample per group was discarded. Finally, ten acceptable DNA samples were delivered to Novogene (Beijing, China) for 16S rDNA sequencing.

The V3-V4 hypervariable region of the bacterial 16S rDNA gene was amplified with the barcoded universal primers (341F-806R). Purified amplicons were sequenced on the Illumina HiSeq platform (Illumina, USA) according to the standard procedures in Novogene (Beijing, China). Sequences with 97% similarity were assigned to the same operational taxonomic units (OTUs). An OTU table was further generated to record the abundance of each OTU in each sample, and a profiling histogram was made using R software (v3.1.1) to represent the relative abundance of taxonomic groups from phylum to species. A Venn diagram was generated to visualize the occurrence of shared and unique OTUs among groups.

### Real-time PCR

Total RNA of tissues in colon, liver, *longissimus dorsi*, subcutaneous fat, and leaf lard were isolated and reversed transcribed to cDNA as previously described (5). The two-step qRT-PCR reactions were performed in triplicate on 96-well plates using a 7500 Real-time PCR system (Applied Biosysytems, Foster, CA) with the SYBR^?^ Premix Ex Taq^TM^ (TaKaRa Biotechnology (Dalian), China). The primer sequences (Table 2) for G protein-coupled bile acid receptor (*TGR*5), farnesoid X receptor (*FXR*), glucagon-like peptide1 (*GLP*1), peptide tyrosine tyrosine (*PYY*), G protein-coupled receptor 41 or 43 (*GPR41, GPR43*), monocarboxylate transporter (*MCT1*), sodium-dependent monocarboxylate transporter (*SMCT1*), lipoprotein lipase (*LPL*), carnitine palmitoyltransferase (*CPT1*), angiopoietin-like protein 4 (*Angptl4*), cluster of differentiation 36 (*CD36*), peroxidase proliferative receptor γ (*PPAR*γ), triglyceride hydrolase (*TGH*), adipose triglyceride lipase (*ATGL*), adipocyte determination and differentiation-dependent factor 1 (*ADD1*), hormone sensitive lipase (*HSL*), fatty acid synthetase (*FAS*), adenosine monophosphate activated protein kinase (*AMP*K) and *GAPDH* were synthesized by the Sangon Biotech (Shanghai, China). Target gene expression was calculated by the 2^−Δ*Δt*^ method relative to *GAPDH* gene amplification.

**Table 2:** Primers for target genes.

**Gene**	**Accession number**	**Sequences (5'−3')**	**Product size (pb)**
*TGR5*	XM_013984487.2	F: CCATGCACCCCTGTTGCT	100
		R: GGTGCTGTTGGGTGTCATCTT	
*FXR*	NM_001287412.1	F: GGTCCTCGTAGAATTCACAA	109
		R: TGAACGGAGAAACATAGCTT	
*GLP1*	NM_214324.1	F: ATGAGGCCGGAACGGAGCCG	140
		R: CTAATCTCACTCTCCTCGAG	
*PPY*	NM_001256528.1	F: AGATATGCTAATACACCGAT	132
		R: CCAAACCCTTCTCAGATG	
*GPR41*	NM_001315601.1	F: TGGAGACCTTACGTGTTG	151
		R: CGAGGATGAGAAGTAGTAGAT	
*GPR43*	NM_001278758.1	F: CGTGTTCATCGTTCAGTA	193
		R: GAAGTTCTCATAGCAGGTA	
*MCT1*	AM286425.1	F: CATCAACTACCGACTTCTG	106
		R: TACTGGTCTCCTCCTCTT	
*SMCT1*	NM_001128445.1	F: CGCAGATTCCTACTAACC	132
		R: GATTGTCAGTTCCACCAT	
*LPL*	NM_214286.1	F: CACATTCACCAGAGGGTC	126
		R: TCATGGGAGCACTTCACG	
*CPT1*	NM_001129805.1	F: GACAAGTCCTTCACCCTCATCGC	117
		R: GGGTTTGGTTTGCCCAGACAG	
*Angptl4*	NM_001038644.1	F: GACTGCCAAGAGCTGTTTGAAGA	173
		R: GACTGCCAAGAGCTGTTTGAAGA	
CD36	NM_001044622.1	F: GCTAGACATCGGCAAATGCAA	133
		R: AGCCTTCAATCGGTCCTGAGA	
*PPARγ*	NM_214379.1	F: ACTTTATGGAGCCCAAGTTC	108
		R: GCAGCAAATTGTCTTGAATGTCC	
*TGH*	NM_214246.2	F: TGAAGAACACCACCTCCTACC	108
		R: CCCTGTGCTGAAGAATCCC	
*ATGL*	NM_001098605.1	F: TCACCAACACCAGCATCCA	129
		R: GCACATCTCTCGAAGCACCA	
*ADD1*	XM_013978492.2	F: GGTAGTGGACACTGACAAGCT	110
		R: GCTTAGCTCAACAGACGGAG	
*HSL*	NM_214315.3	F: GCCTTCTCAGCCTCATGGACCC	119
		R: CCGCACCAGTCCCATCATGCC	
*FAS*	NM_213839.1	F: TTTTCCCTGGCACTGGCTACCTG	81
		R: TGCAGCGTCACGTCCTCAAACAC	
*AMPK*	NM_001167633.1	F: ACGACGGGCGGGTGAAAATCG	141
		R: CACTTTGCCGAAGGTCCCGACC	
*GAPDH*	NM_001206359.1	F: ATGGTGAAGGTCGGAGTGAAC	235
		R: CTCGCTCCTGGAAGATGGT	

TGR5, G protein-coupled bile acid receptor; FXR, Farnesoid X receptor; GLP1, Glucagon-like peptide; PYY, peptide tyrosine tyrosine; GPR41, G protein-coupled receptor41; GPR43, G protein-coupled receptor43; MCT1, Monocarboxylate transporter; SMCT1, Sodium-dependent monocarboxylate transporter; LPL, Lipoprotein lipase; CPT1, Carnitine palmitoyltransferase; Angptl4, Angiopoietin-like protein 4; CD36, Cluster of differentiation 36; PPARγ, Peroxidase proliferative receptor γ; TGH, Triglyceride hydrolase; ATGL, Adipose triglyceride lipase; ADD1, Adipocyte determination and differentiation-dependent factor 1; HSL, Hormone sensitive lipase; FAS, Fatty acid synthetase; AMPK, Adenosine monophosphate activated protein kinase.

### Determination of TG concentrations in selected tissue

The contents of TG (mmol/g.prot) in the liver, *longissimus dorsi*, subcutaneous fat or leaf lard was measured according to our previous description (5).

### Statistical analysis

All results were expressed as Mean ± SD. Statistical analysis, except for microbiotal data, were conducted by the two-tailed unpaired Student's *t*-test of SPSS 17.0 (SPSS Inc., Chicago, IL, USA), with individual pig as an experimental unit. The Kruskal test was used for *post-hoc* comparison of taxonomy. For all tests, *P* < 0.05 was considered as significant difference, while 0.05 < *P* < 0.10 as a tendency.

## Results

All pigs appeared in good health during the 28-d trial. Administration of *L. delbrueckii* into diet did not significantly (*P* > 0.05) affect the growth performance of growing-finishing pigs, and the detailed results have been reported in our previous study (5, Supplementary Table 1).

### Serum lipids and hormone levels

**Figure 1:**
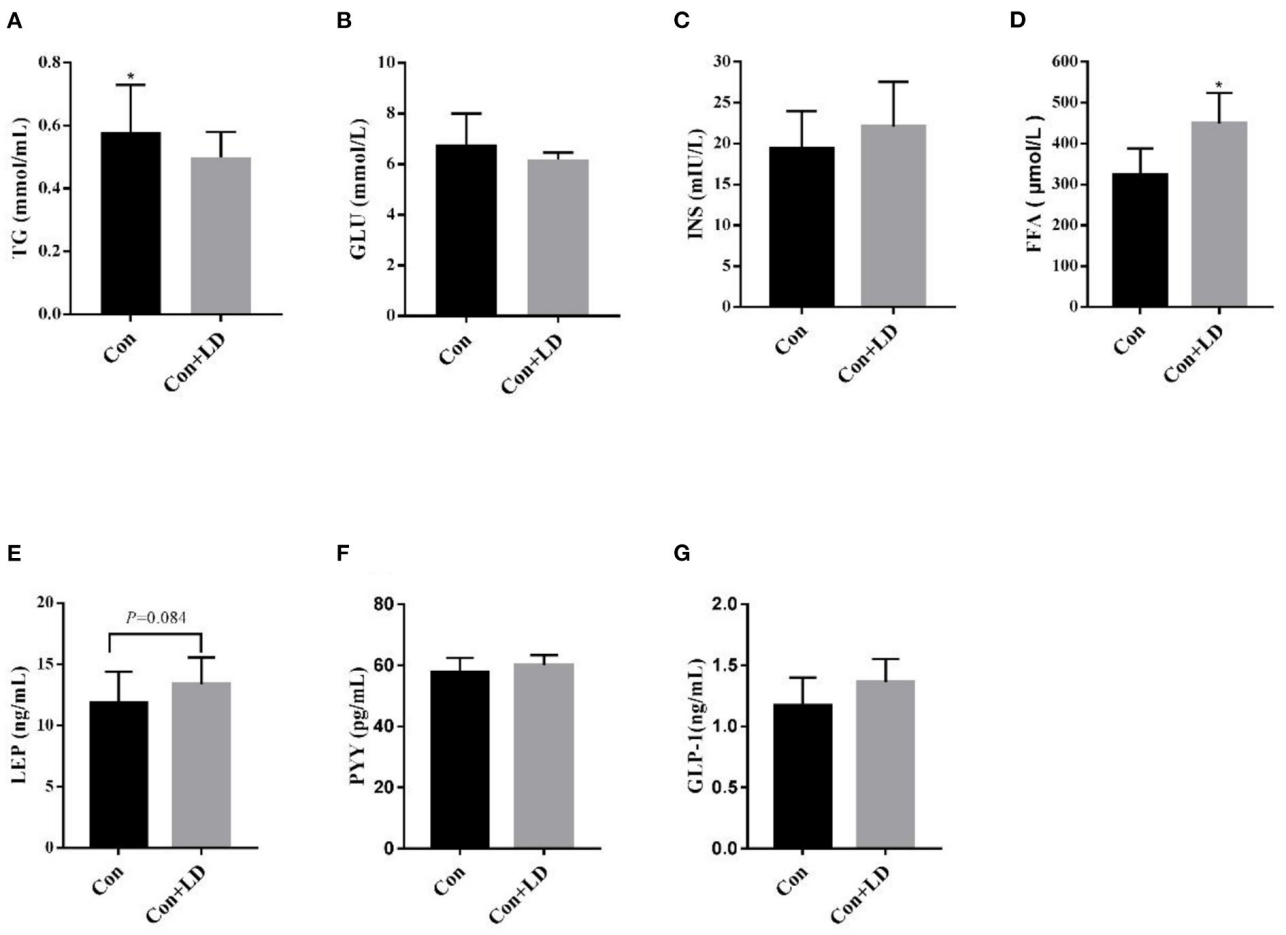
Serum levels of lipids and hormone in growing-finishing pigs. Values for histogram are shown as Mean ± SEM, *P < 0.05. **(A)** TG, triglyceride; **(B)** GLU, glucose; **(C)** INS, insulin; **(D)** FFA, free fatty acids; **(E)** LEP, leptin; **(F)** PYY, peptide tyrosine tyrosine; **(G)** GLP-1, glucagon-likepeptide-1; Con, basal diet; Con + LD, basal diet + 0.1 % *L. delbrueckii*.

Dietary *L. delbrueckii* significantly lowered (*P* < 0.05) serum TG levels (Figure 1A), elevated (*P* < 0.05) serum contents of FFA (Figure 1D) and tended to increase (*P* = 0.084) serum LEP concentrations (Figure 1E) compared with the control group. No significant differences were found between two groups regarding serum levels of GLU, INS, PPY and GLP-1 (Figures 1B,C,F,G, *P* > 0.05).

### Serum and colonic SCFA composition

Butyric acid concentrations in colonic digesta in the Con+LD group was higher than those in the Con group (Table 3, *P* < 0.05). Meanwhile, serum contents of butyric acid in the Con+LD group tended to elevate compared with the Con group (*P* = 0.064). However, no obvious differences were observed in other detected SCFAs between two groups (*P* > 0.05).

**Table 3:** Effects of *L. delbrueckii* on SCFA composition in the serum and colonic digesta of growing-finishing pigs.

**Items**	**Con**	**Con+LD**	***P*-value**
Serum (μmol/L)
Acetic acid	161.27 ± 16.58	173.97 ± 37.21	0.247
Propionic acid	47.03 ± 7.21	52.02 ± 9.02	0.143
Butyric acid	1.73 ± 0.07	2.07 ± 0.09	0.064
Total SCFAs	210.04 ± 20.23	228.06 ± 31.08	0.835
Colonic digesta (mg/g)
Acetic acid	1.56 ± 0.19	1.71 ± 0.27	0.076
Propionic acid	0.67 ± 0.08	0.82 ± 0.02	0.093
Butyric acid	0.53 ± 0.06	0.76 ± 0.09*	0.042
Total SCFAs	2.76 ± 0.14	3.31 ± 0.24	0.131

Con, basal diet; Con + LD, basal diet + 0.1 % L. delbrueckii; SCFA, Short chain fatty acids. *Means significant difference (*P* < 0.05).

### Colonic bacterial structure

**Figure 2:**
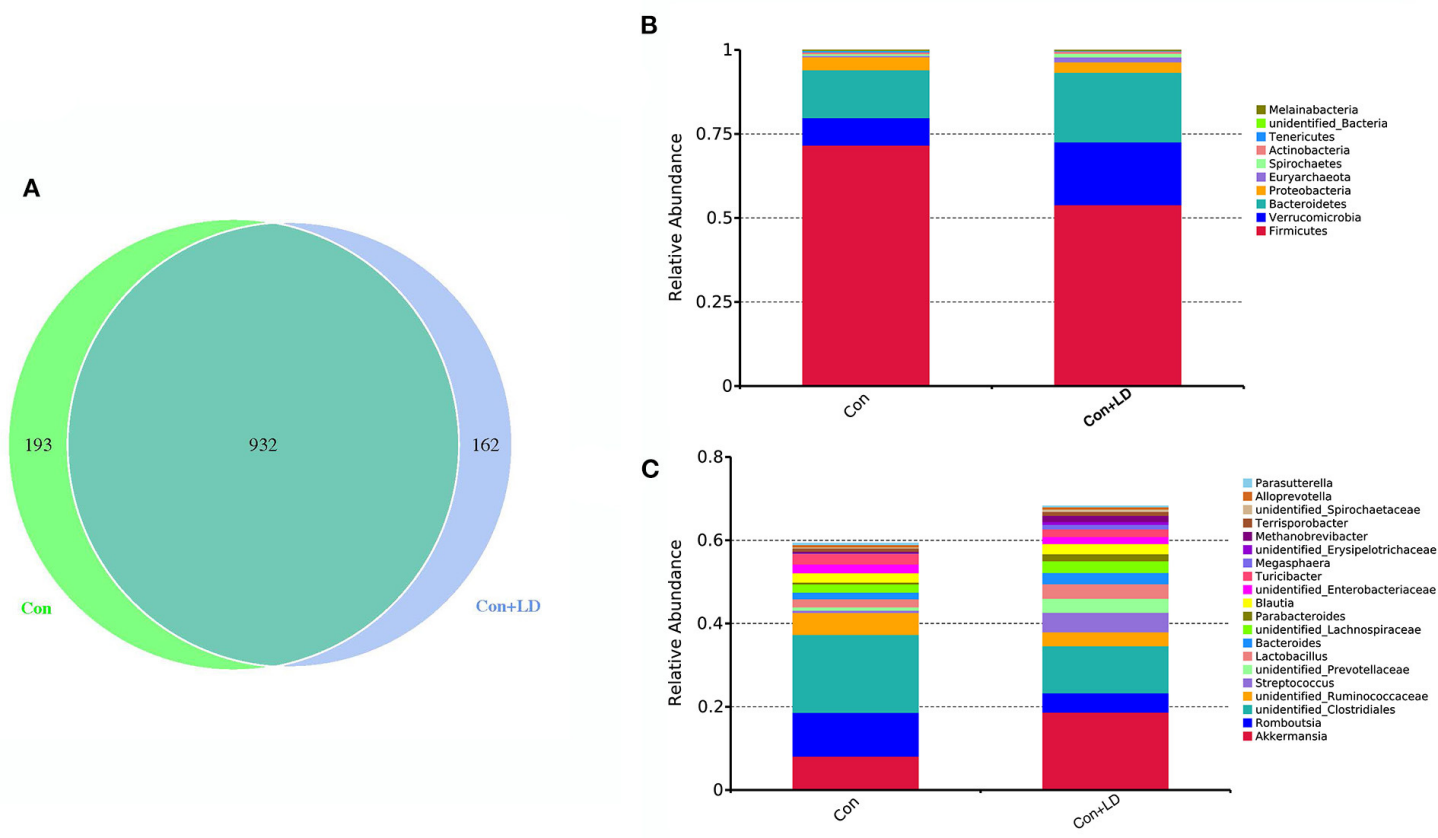
Colonic bacterial composition of growing-finishing pigs. **(A)** Venn picture showed shared or unique OTUs between two groups. Abundant phyla **(B)** and genera **(C)** in the colonic microbiota. Con, basal diet; Con + LD, basal diet + 0.1 % *L. delbrueckii*.

The Venn diagram exhibited 932 shared OTUs between two groups, and 193 and 162 particular OTUs were seen in the Con and Con + LD group, respectively (Figure 2A). At phylum level, Firmicutes, Bacteroidetes, Actinobacteria and Proteobacteria were four dominant bacterial population (Figure 2B). Administration of *L. delbrueckii* increased Euryarchaeota and Spirochaetes abundance (*P* < 0.01), but tended to lower the abundance of Firmicutes (*P* = 0.092) and Melainabacteria (*P* = 0.096) and the Firmicutes/ Bacteroidetes ratio (Figure 2B and Supplementary Table 2). Down to the genus level, higher abundance of *Lactobacillus, Erysipelotrichaceae, Methanobrevibacter, Spirochaetaceae* and *Butyrivibri*, and lower abundance of *Romboutsia, Clostridiales* and *Streptococcus* were seen in the Con + LD group (*P* < 0.05, Figure 2C and Supplementary Table 2). The abundance of *Akkermansia* (*P* = 0.060), *Parabacteroides* (*P* = 0.099), *Turicibacter* (*P* = 0.074), and *Megasphaera* (*P* = 0.077) tended to increase, but *Ruminococcaceae* (*P* = 0.065) tended to decrease in the Con + LD group.

### Colonic gene expression associated with SCFA transport and signaling

**Figure 3:**
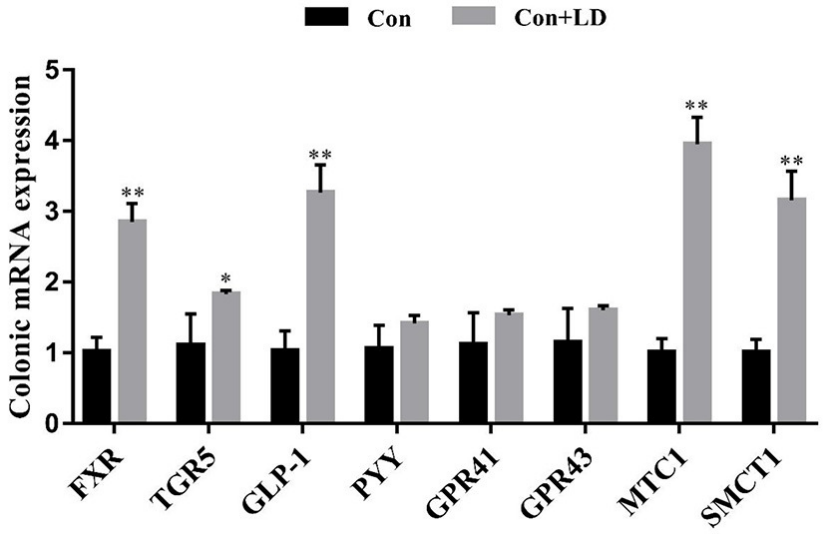
Colonic gene expression related to SCFAs transport and signal transduction of growing-finishing pigs. Values for histogram are shown as Mean ± SEM, *P < 0.05, **P < 0.01. *FXR*, Farnesoid X receptor; *TGR5*, G protein-coupled bile acid receptor; *GLP1*, Glucagon-like peptide 1; *PPY*, Peptide YY; *GPR41*, G protein-coupled receptor41; *GPR43*, G protein-coupled receptor43; *MCT1*, Monocarboxylate transporter; *SMCT1*, Sodium-dependent monocarboxylate transporter; Con, basal diet; Con + LD, basal diet + 0.1 % *L. delbrueckii*.

Several genes involved in SCFA transport and signaling of colon tissues were measured using qRT-PCR (Figure 3). *L. delbrueckii* consumption led to up-regulated *FXR, TGR5, GLP-1, MTC1* and *SMCT1*expression compared to the control group (*P* < 0.05). The mRNA expression of remaining target genes in colon were no differences between two treatments (*P* > 0.05).

### Lipid metabolism related enzyme activity and gene expression in liver

**Figure 4:**
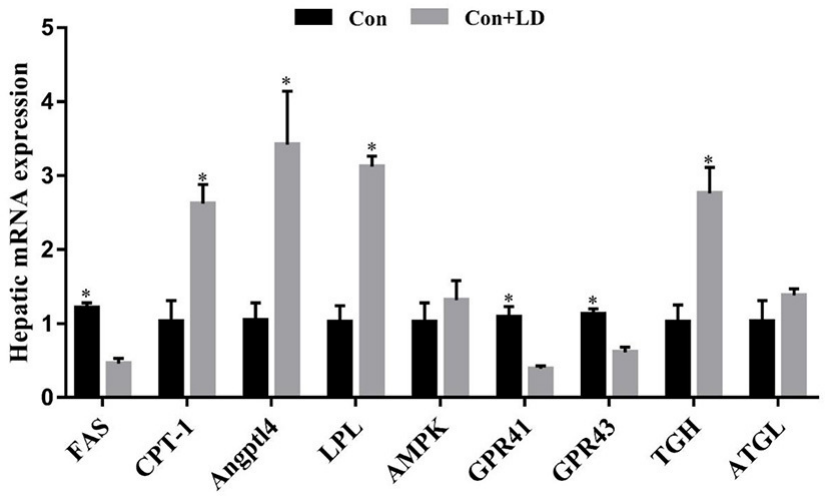
Hepatic gene expression related to lipid metabolism of growing-finishing pigs. Values for histogram are shown as Mean ± SEM, *P < 0.05. *FAS*, Fatty acid synthetase; *CPT1*, Carnitine palmitoyltransferase 1; *Angptl4*, Angiopoietin-like protein 4; *LPL* = Lipoprotein lipase; *AMPK* = Adenosine monophosphate activated protein kinase; *GPR41*, G protein-coupled receptor41; *GPR43*, G protein-coupled receptor43; *TGH*, Triglyceride hydrolase; *ATGL*, Adipose triglyceride lipase; Con = basal diet; Con + LD, basal diet + 0.1 % *L. delbrueckii*.

*L. delbrueckii* addition tended to increase LPL activity (*P* = 0.056), however, did not affect (*P* > 0.05) other selected lipid metabolism related enzyme activity compared with the control diet (Table 4). Dietary *L. delbrueckii* down-regulated (*P* < 0.05) *FAS, GPR41* and *GPR43* expression, but up-regulated (*P* < 0.05) *CPT-1, Angpt14, LPL* and *TGH* expression relative to the Con group (Figure 4).

**Table 4:** Effects of *L. delbrueckii* on hepatic enzyme activity related to fat metabolism of growing-finishing pigs.

**Items**	**Con**	**Con+LD**	***P*-value**
HSL (U/g.prot)	207.89 ± 64.38	254.68 ± 40.18	0.167
LPL (U/g.prot)	158.51 ± 23.02	180.12 ± 41.12	0.056
FAS (U/g.prot)	266.40 ± 85.76	286.57 ± 76.98	0.906
ATGL (IU/g.prot)	74.41 ± 13.24	63.77 ± 9.70	0.293

Con, basal diet; Con + LD, basal diet + 0.1 % L. delbrueckii; HSL, Hormone sensitive lipase; LPL, Lipoprotein lipase; FAS, Fatty acid synthetase; ATGL, Adipose triglyceride lipase.

### Gene expression related to lipid metabolism in muscle and fat tissues

**Figure 5:**
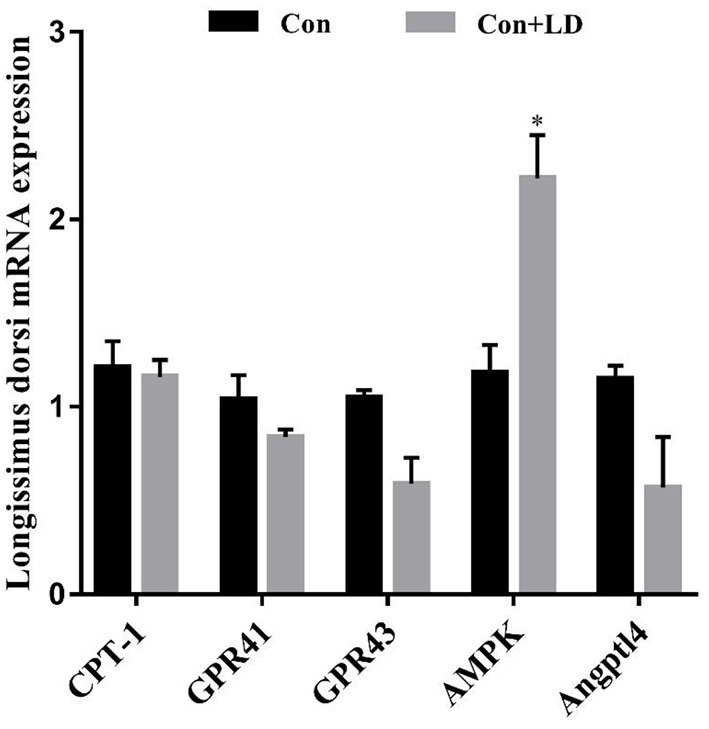
Gene expression related to lipid metabolism in *longissimus dorsi* of growing-finishing pigs. Values for histogram are shown as Mean ± SEM, *P < 0.05. *CPT1*, Carnitine palmitoyltransferase 1; *GPR41*, G protein-coupled receptor41; *GPR43*, G protein-coupled receptor43; *AMPK*, Adenosine monophosphate activated protein kinase; *Angptl4*, Angiopoietin-like protein 4; Con, basal diet; Con + LD, basal diet + 0.1 % *L. delbrueckii*.

**Figure 6:**
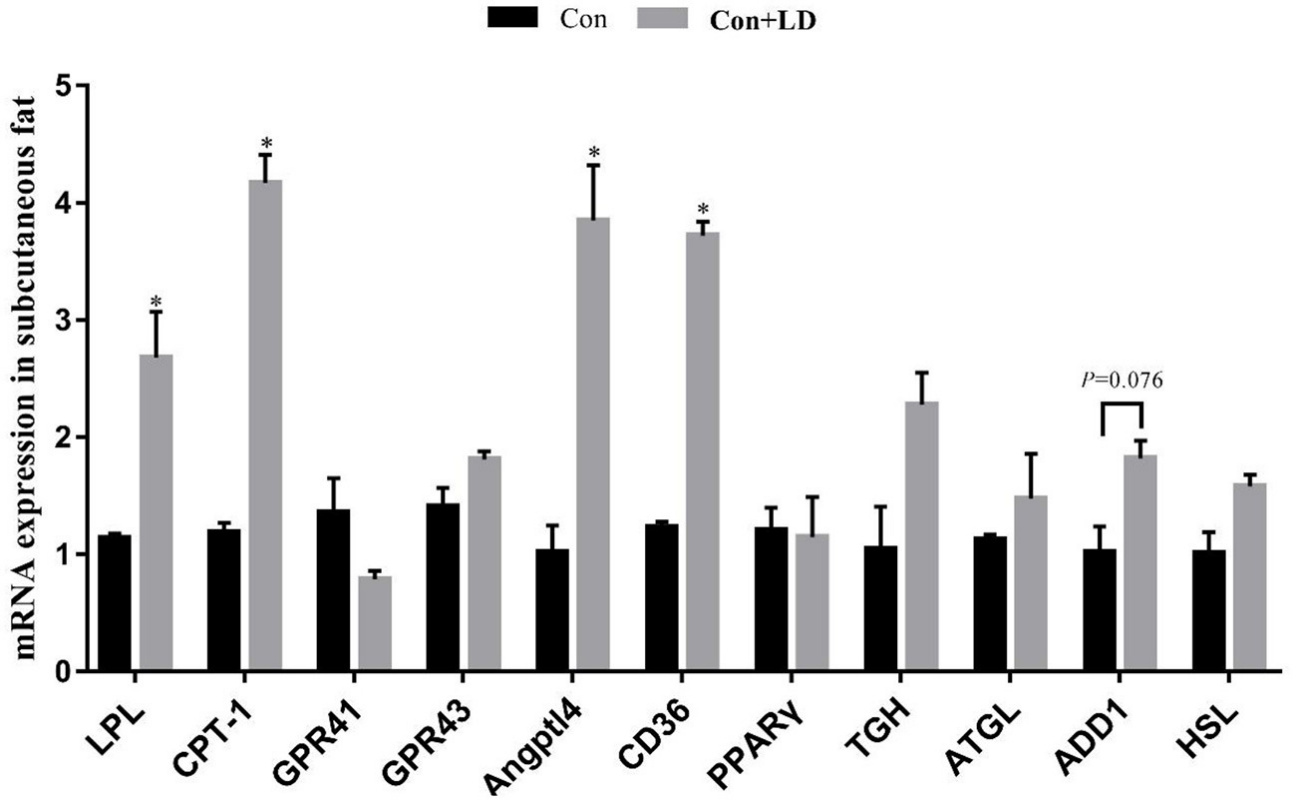
Gene expression related to lipid metabolism in subcutaneous fat of growing-finishing pigs. Values for histogram are shown as Mean ± SEM, *P < 0.05. *LPL*, Lipoprotein lipase; *CPT1*, Carnitine palmitoyltransferase 1; *GPR41*, G protein-coupled receptor41; *GPR43*, G protein-coupled receptor43; *Angptl4*, Angiopoietin-like protein 4; *CD36*, Cluster of differentiation 36; *PPAR*γ, Peroxidase proliferative receptor γ; *TGH*, Triglyceride hydrolase; *ATGL*, Adipose triglyceride lipase; *ADD1*, Adipocyte determination and differentiation-dependent factor 1; *HSL*, Hormone sensitive lipase; Con, basal diet; Con + LD, basal diet + 0.1 % *L. delbrueckii*.

**Figure 7:**
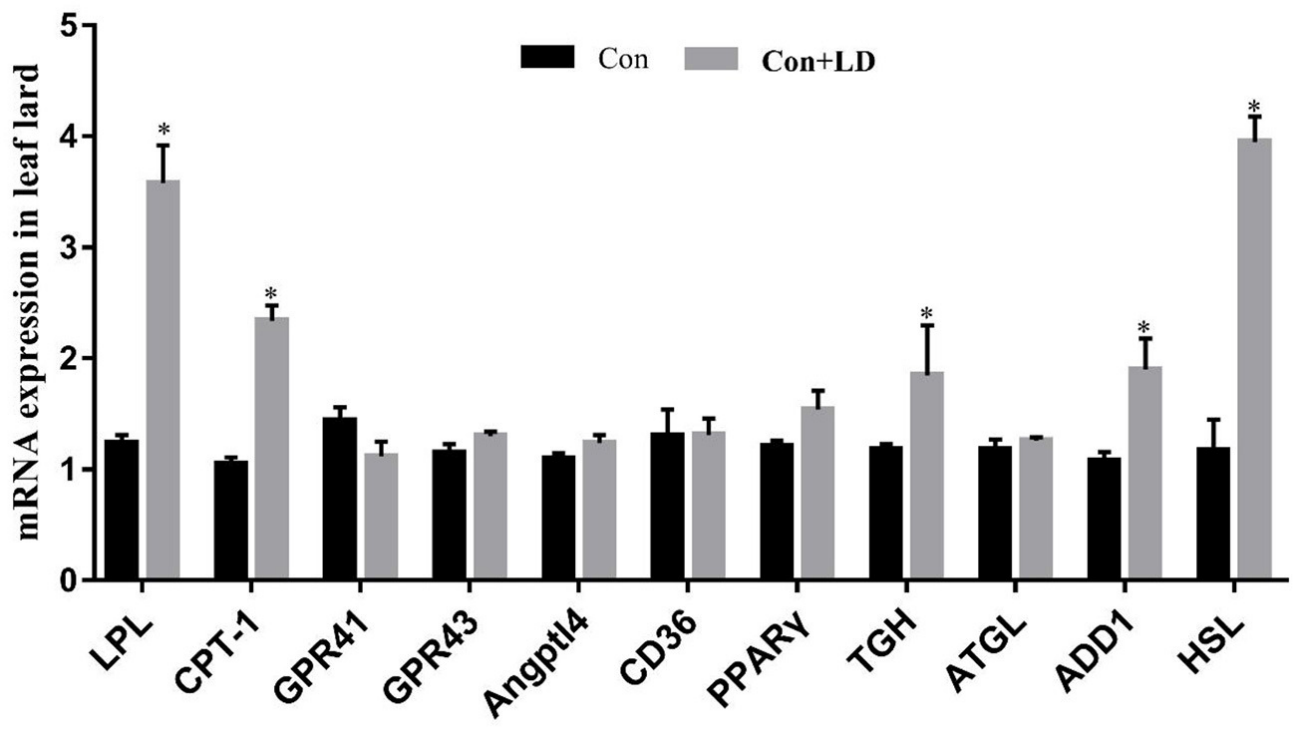
Gene expression related to lipid metabolism in leaf lard of growing-finishing pigs. Values for histogram are shown as Mean ± SEM, *P < 0.05. *LPL*, Lipoprotein lipase; *CPT1*, Carnitine palmitoyltransferase 1; *GPR41*, G protein-coupled receptor41; *GPR43*, G protein-coupled receptor43; *Angptl4*, Angiopoietin-like protein 4; *CD36*, Cluster of differentiation 36; *PPAR*γ, Peroxidase proliferative receptor γ; *TGH*, Triglyceride hydrolase; *ATGL*, Adipose triglyceride lipase; *ADD1*, Adipocyte determination and differentiation-dependent factor 1; *HSL*, Hormone sensitive lipase; Con, basal diet; Con + LD, basal diet + 0.1 % *L. delbrueckii*.

Administration of *L. delbrueckii* increased *AMPK* expression in *longissimus dorsi* (Figure 5, *P* < 0.05). Expression of *LPL, CPT-1, Angptl4* and *CD36* were upregulated (*P* < 0.05), and there was a upward trend for *ADD*1 expression (*P* = 0.076) in subcutaneous fat by *L. delbrueckii* addition (Figure 6). Dietary *L. delbrueckii* enhanced *LPL, CPT-1, TGH, ADD1* and *HSL* expression in leaf lard (Figure 7, *P* < 0.05).

### Concentrations of TG in various tissue

Dietary *L. delbrueckii* did not affect the TG contents in the selected tissue, and the detailed results are showed in our previous study (5, Supplementary Table 3).

## Discussion

Lipid control by probiotics in animal or human studies have attracted extensive attention, and increasing evidences manifested probiotics consumption can effectively reduce serum TG, TC and LDL-C levels (25). *Lactobacillus* is the most popular probiotic and a meta-analysis of *Lactobacillus* (*L*.reuteri and *L*.plantarum) lowering serum TG, TC and LDL-C is reviewed by Wu et al. (15). Our results showed dietary *L. delbrueckii* decreased serum TG contents, elevated serum FFA concentrations and tended to increase serum LEP levels of growing-finishing pigs, coinciding with our previous report (5, 20). The reduced serum TG and increased serum FFA might ascribe the catabolism of TG to produce FFA and glycerol, because TG are first hydrolyzed to diacylglycerol (DG), then to monoacylglycerol (MG), and finally to FFA and glycerol under the action of ATGL, HSL and Monoacylglycerol lipase (MGL), respectively (26). LEP is an adipose tissue-derived protein hormone, participating in glucose and lipid metabolism via inhibition of feeding and fat synthesis and promotion of energy expenditure (27–29). The elevated trend of serum LEP might imply pigs had low feedintake and fat deposition but high energy consumption after *L. delbrueckii* treatment. Gastrointestinal tract is the main site where microbiota colonize and inhabit (29). Intestinal microbes are dense bioactive communities serving as the junction between animals and their nutritional environment and their activity profoundly affects many aspects of host animal physiology and metabolism (30). These alterations in serum levels of lipids and hormone might be associated with the changes of intestinal microbiota and metabolites of pigs after *L. delbrueckii* consumption.

The swine gut harbors vast, highly diverse and dynamic microbial communities exhibiting a vital role in the host nutrient acquisition and energy homoeostasis (23, 31, 32). Our findings showed that the abundance of Firmicutes and the Firmicutes to Bacteroidetes (F/B) ratio tended to reduce after *L. delbrueckii* administration. The increased Firmicutes abundance, lowered Bacteroidetes abundance and the enhanced F/B ratio were positively associated with the individual obesity (33). Obesity primarily results from imbalance of energy intake and expenditure (9). Changes at the level of the phylum indicated dietary *L. delbrueckii* might interfere with the energy metabolism and ameliorate obesity. Down to the genus level, the abundance of *Akkermansia, Lactobacillus, Parabacteroides, Turicibacter, Megasphaera, Methanobrevibacter* and *Butyrivibrio* were elevated by *L. delbrueckii* addition. *Akkermansia* is a mucin-degrading bacterium colonizing in the mucus and inversely correlates with body weight in rodents and humans (34), and now it is recommended as a new probiotic to deal with obesity, diabetes and liver disease (35). *Lactobacillus* has been widely applied in animal production, and the increased *Lactobacillus* abundance in the colonic digesta might be main the *L. delbrueckii*, which has been proven probiotic roles in promoting intestinal nutrients digestion and absorption and regulating lipid metabolism of pigs in our previous studies (5, 21, 36). *Parabacteroides distasonis* can alleviate obesity and metabolic dysfunctions via production of succinate and secondary bile acids (37). *Turicibacter* can produce lactic acid during fermentation and play a crucial role on muscle regulation and anti-fatigue (37). *Megasphaera* and *Butyrivibrio* belongs to butyrate-producing bacteria (38), and butyrate has received particular attention for its beneficial effects on intestinal homeostasis and energy metabolism (39). These results indicated dietary *L. delbrueckii* might be tightly link to the host energy and lipid metabolism via colonic microbiota modulation.

The gut microbiota can participate in host lipid metabolism through their metabolites, such as SCFAs, secondary bile acids, trimethylamine and lipopolysaccharide (40). SCFAs are mainly generated by colon fermentation and more than 95% of SCFAs are rapidly absorbed and transported into blood, and subsequently are taken up and metabolized by body organs as substrates or signal molecules, participating in energy and lipid metabolism (12, 13, 41–43). For example, butyrate is almost completely used by colonocytes as their preferred energy substrate, whereas acetate and propionate move to the liver *via* the portal vein and are used for cholesterol and fatty acid synthesis and gluconeogenesis, respectively (13). In our study, the concentrations of acetic, propionic and butyric acid as well as total SCFA in colonic digesta rose in different extent in the Con + LD group. The elevated concentrations of butyrate might be associated with the increased abundance of *Megasphaera* and *Butyrivibrio* aforementioned. Meanwhile, the mRNA expression of SCFA transporters (*MTC*1, *SMCT*1) were upregulated might be due to the increased SCFA contents. Furthermore, activation of *FXR*α and *TGR*5 can improve triglyceride control via stimulating thyroid hormone in brown adipose tissue and muscle to increase energy expenditure (15). PYY and GLP-1 are two anorectic gut hormones secreted from enteroendocrine L-cells, and the upregulated colonic *GLP*-1 illustrated the satiety signal was amplified, coinciding with the change of serum LEP level, which might be link to the appetite regulation of SCFAs (44, 45). Additionally, the values for contents of corresponding serum SCFA profiles showed the similar tendency as those in colonic digesta, which implied that SCFA produced in colon did not completely used onsite and the remaining entered into the blood to take part in whole body metabolism. The findings implied that SCFAs might be an important bridge between gut microbiota and parenteral tissues or organs in host metabolism.

The liver is the center of lipid synthesis and metabolism. FAS, HSL, LPL and ATGL are the key enzymes for hepatic fatty acid synthesis and lipolysis (46). FAS is responsible for lipogenesis, whereas HLS, LPL and TGH are in charge of lipolysis. In our study, dietary *L. delbrueckii* tended to increase the LPL enzyme activity, upregulated *LPL* and *TGH* mRNA expression and down-regulated *FAS* mRNA expression in the liver, illustrating hepatic lipolysis was strengthened but lipogenesis weakened. LPL provides fatty acid for tissue utilization and storage and can be inhibited by Angptl4. Angptl4 is highly expressed in liver and adipose tissue and susceptible to regulation by gut microbiota and SCFAs, controlling TG deposition into adipocytes (11, 47). However, we found the *Angptl4* and *LPL* expression were both increased, which might be related to the SCFAs generated in colon, just as a report from Jiao et al. (45) that SCFAs could reduce lipogenesis, and enhance lipolysis in different tissues of pigs via regulating related hormones and genes. CPT1 is a rate-limiting enzyme in the β-oxidation process of fatty acids and located in the outer mitochondrial membrane, catalyzing the transfer of long-chain fatty acids from acyl-CoA to carnitine, then it enters the mitochondria from the cytoplasm for oxidation (14). The upregulated hepatic *CPT-1* mRNA expression demonstrated that fatty acid β-oxidation might be enhanced. The aboved description indicated that the decreased lipogenesis and increased lipolysis in the liver might be due to the SCFA-mediated regulation of related genes and enzyme activity.

Skeletal muscle and adipose tissue are important sites involved in fatty acid metabolism. Our results showed that mRNA expression of *AMPK* in longissimus, *LPL, CPT*-1, *Angptl*4, *CD*36 and *ADD*1 in subcutaneous fat, and *LPL, CPT*-1, *TGH, ADD*1 and *HSL* in leaf lard were upregulated by dietary *L. delbrueckii*. AMPK is a key regulator of energy metabolism, and activated AMPK can promote catabolic pathways, resulting in ATP generation and inhibiting ATP-consumed anabolic pathways to restore body energy homeostasis (48). LPL, TGH, HSL, Angptl4 and CPT-1 involved in lipolysis and fatty acid β-oxidation process as previously mentioned. CD36 is widely distributed on the cell surface, including extracellular, transmembrane and cytoplasmic region, is an important FA transport receptor that mediates FA uptake of tissue, which accounts for 50% of the FA uptake in adipose tissues and muscle in mice (49). ADD1 belongs to a member of SREBPs family, directly involving in fat synthesis and glucose metabolism by regulating gene expression of *PPAR*γ, *FAS, ACC* and *PEPCK* (50). These results implied administration of *L. delbrueckii* might increase lipolysis and energy expenditure in muscle and adipose tissues of growing-finishing pigs via regulation of lipid metabolism related genes.

## Conclusions

**Figure 8:**
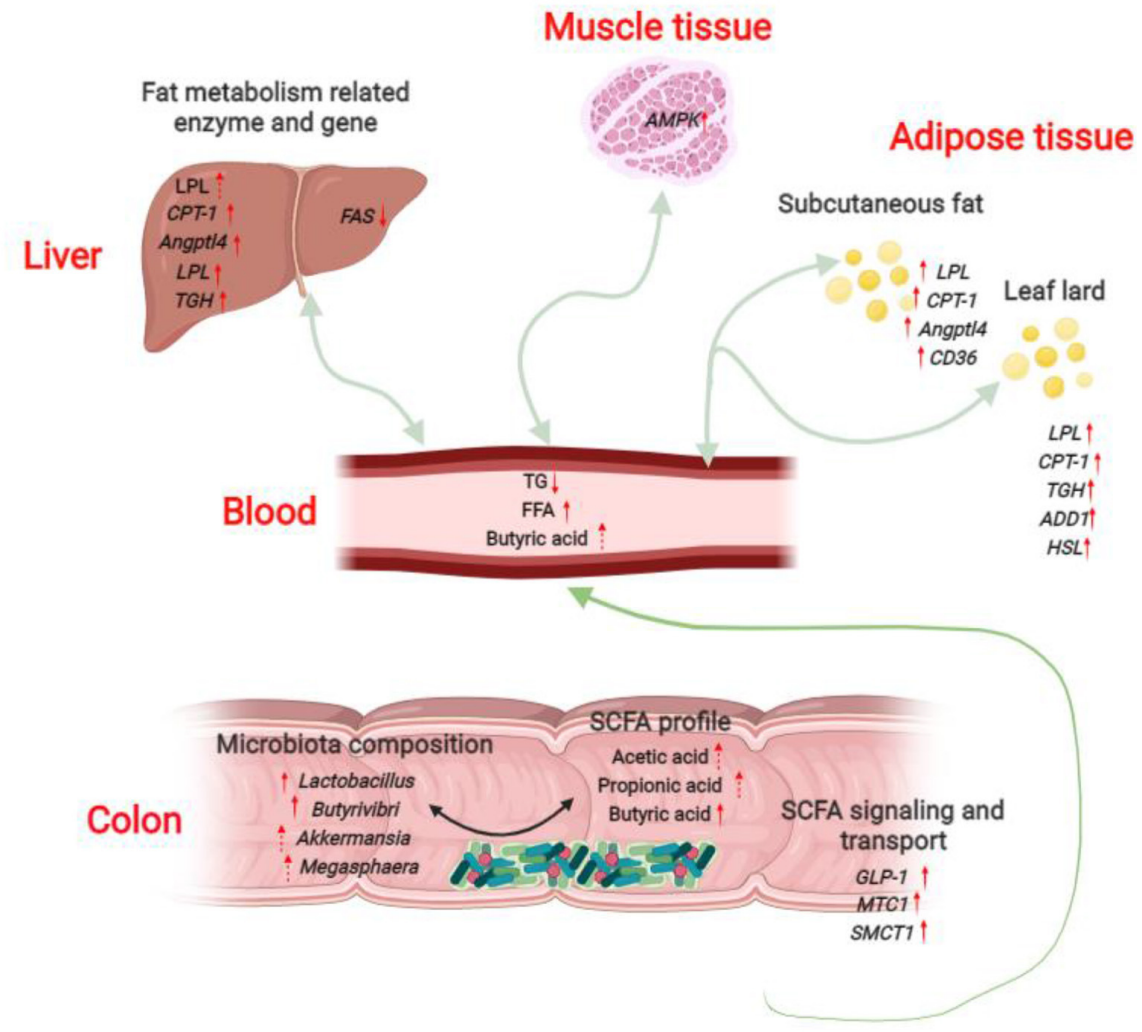
Potential serum triglyceride-lowering mechanisms of *L. delbrueckii -*fed growing-finishing pigs. Colonic microbiota modulation (mainly increased the abundance of *Lactobacillus, Butyrivibri, Akkermansia* and *Megasphaera*) after *L. delbrueckii* administration increased the concentrations of acetic acid, propionic acid and butyric acid. SCFAs produced in colon are rapidly absorbed and transported into blood through the transporters (up-regulation of *MTC*1 and *SMCT*1) and subsequently taken up and metabolized by body organs (Liver, *longissimus dorsi*, subcutaneous fat and leaf lard in the study) as substrates or signal molecules, participating in energy and lipid metabolism. SCFAs might mediate the strengthened lipolysis (increased expression of *LPL, TGH, HSL*) and fatty acid β-oxidation (up-regulation of *CPT*-1 and *AMPK*) and the weakened fat synthesis (down-regulation of *FAS*) in liver, muscle and fat tissues.

Taken together, our results demonstrated that dietary *L. delbrueckii* might lower serum TG levels of growing-finishing pigs via enhancing colonic *Lactobacillus, Butyrivibri, Akkermansia and Megasphaera* abundance and Butyric acid content, and upregulating mRNA expression of genes related lipolysis and fatty acid β-oxidation in liver, muscle and fat tissues (Figure 8).

## Data availability statement

The datasets presented in this study can be found in online repositories. The names of the repository and accession number are SRA and PRJNA670289 respectively.

## Ethics statement

The animal study was reviewed and approved by the Animal Care and Use Committee of the Institute of Subtropical Agriculture, Chinese Academy of Sciences under the permit number IACUC#201302 (Changsha, China). Written informed consent was obtained from the owners for the participation of their animals in this study.

## Author contributions

RL, GH, and XH are responsible for the conceptualization. GH, LW, and YYu contribute to animal trail, sample collection, and measurement. GH and RL are responsible for data analysis and drafting of the manuscript. WP and JY provides technical support. XH, RL, and YYi contribute to the resources. RL, JY, and YYi are responsible for supervision and critical revision of the manuscript. All authors contributed to the article and approved the submitted version.

## Funding

This research was jointly supported by grants from the National Key Research and Development Program of China (2021YFD1300201 and 2021YFD1301004), the National Natural Science Foundation of China (U20A2055 and 32172761), the science and technology innovation Program of Hunan Province (2020RC2063), the Natural Science Foundation of Hunan Province (2022JJ40532), and the Open Fund of Key Laboratory of Agro-ecological Processes in Subtropical Region, Chinese Academy of Sciences (ISA2021103).

## Conflict of interest

The authors declare that the research was conducted in the absence of any commercial or financial relationships that could be construed as a potential conflict of interest.

## Publisher's note

All claims expressed in this article are solely those of the authors and do not necessarily represent those of their affiliated organizations, or those of the publisher, the editors and the reviewers. Any product that may be evaluated in this article, or claim that may be made by its manufacturer, is not guaranteed or endorsed by the publisher.

## References

[1] He YN Yang XG Xia J Zhao LY Yang YX. Consumption of meat and dairy products in China: a review. Proc Nutr Soc. (2016) 75:385–91. 10.1017/S0029665116000641 27334652

[2] USDA. Livestock and Poultry: World Markets and Trade, China Meat Consumption Expected to Rebound in 2021. USDA (2021). Available online at: https://downloads.usda.library.cornell.edu/usda-esmis/files/73666448x/t435h4995/vt1519922/livestock_poultry.pdf (accessed January 12, 2021).

[3] Wu L Gong X Chen X Hu W. Compromise effect in food consumer choices in china: an analysis on pork products. Front Psychol. (2020) 11:1352. 10.3389/fpsyg.2020.01352 32695046 PMC7339376

[4] Lim MSM Grohn YT. Comparison of China's and the European Union's approaches to antimicrobial stewardship in the pork industry. Foodborne Pathog Dis. (2021) 18:567–73. 10.1089/fpd.2020.2887 33794668

[5] Hou G Peng W Wei L Li R Yuan Y Huang X. *Lactobacillus delbrueckii* interfere with bile acid enterohepatic circulation to regulate cholesterol metabolism of growing-finishing pigs via its bile salt hydrolase activity. Front Nutr. (2020) 7:617676. 10.3389/fnut.2020.617676 33363199 PMC7759492

[6] Godfray HCJ Aveyard P Garnett T Hall JW Key TJ Lorimer J. Meat consumption, health, and the environment. Science. (2018) 361:eaam5324. 10.1126/science.aam5324 30026199

[7] Xu K Ji M Huang X Peng YJ Wu WJ Zhang J. Differential regulatory roles of micrornas in porcine intramuscular and subcutaneous adipocytes. J Agr Food Chem. (2020) 68:3954–62. 10.1021/acs.jafc.9b08191 32146812

[8] Ma J Duan YH Li R Liang XX Li TJ Huang XG. Gut microbial profiles and the role in lipid metabolism in shaziling pigs. Anim Nutr. (2022) 9:345–56. 10.1016/j.aninu.2021.10.012 35600540 PMC9111993

[9] Joyce SA MacSharry J Casey PG Kinsella M Murphy EF Shanahan F. Regulation of host weight gain and lipid metabolism by bacterial bile acid modification in the gut. Proc Natl Acad Sci U S A. (2014) 111:7421–6. 10.1073/pnas.1323599111 24799697 PMC4034235

[10] Nieuwdorp M Gilijamse PW Pai N Kaplan LM. Role of the microbiome in energy regulation and metabolism. Gastroenterology. (2014) 146:1525–33. 10.1053/j.gastro.2014.02.008 24560870

[11] Cui C Shen CJ Jia G Wang KN. Effect of dietary bacillus subtilis on proportion of bacteroidetes and firmicutes in swine intestine and lipid metabolism. Genet Mol Res. (2013) 12:1766–76. 10.4238/2013.May.23.1 23765983

[12] Wu C Lyu W Hong Q Zhang X Yang H Xiao Y. Gut microbiota influence lipid metabolism of skeletal muscle in pigs. Front Nutr. (2021) 8:675445. 10.3389/fnut.2021.675445 33928112 PMC8076524

[13] Canfora EE Jocken JW Blaak EE. Short-chain fatty acids in control of body weight and insulin sensitivity. Nat Rev Endocrinol. (2015) 11:577–91. 10.1038/nrendo.2015.128 26260141

[14] Yi R Tan F Zhou X Mu J Li L Du X. Effects of lactobacillus fermentum cqpc04 on lipid reduction in c57bl/6j mice. Front Microbiol. (2020) 11:573586. 10.3389/fmicb.2020.573586 33013810 PMC7494803

[15] Wu Y Zhang Q Ren Y Ruan Z. effect of probiotic lactobacillus on lipid profile: a systematic review and meta-analysis of randomized, controlled trials. PLoS ONE. (2017) 12:e0178868. 10.1371/journal.pone.0178868 28594860 PMC5464580

[16] Tian Z Cui Y Lu H Wang G Ma X. Effect of long-term dietary probiotic lactobacillus reuteri 1 or antibiotics on meat quality, muscular amino acids and fatty acids in pigs. Meat Sci. (2021) 171:108234. 10.1016/j.meatsci.2020.108234 32906013

[17] Hao P Zheng HJ Yu Y Ding GH Gu WY Chen ST. Complete sequencing and pan-genomic analysis of lactobacillus delbrueckii subsp bulgaricus reveal its genetic basis for industrial yogurt production. Plos ONE. (2011) 6:e15964. 10.1371/journal.pone.0015964 21264216 PMC3022021

[18] Wang XL Liu ZY Li YH Yang LY Yin J He JH. Effects of dietary supplementation of lactobacillus delbrueckii on gut microbiome and intestinal morphology in weaned piglets. Front Vet Sci. (2021) 8:692389. 10.3389/fvets.2021.692389 34490392 PMC8417114

[19] Hou G Li R Liu M Peng W Pan J Li S. Effects of *Lactobacillus delbrueckii* on carcass traits and meat quality of fattening pigs. Chin J Anim Nutr. (2016) 28:1814–22. 10.3969/j.issn.1006-267x.2016.06.024

[20] Li R Hou G Wei L Peng W Pan J Huang X. Effects of *Lactobacillus delbrueckii* on serum biochemical parameters, related genes mRNA expression of cholesterol metabolism and fat deposition in finishing pigs. Chin J Anim Nutr. (2017) 29:3184–92. 10.3969/j.issn.1006-267x.2017.09.021

[21] Wei L Li R Liu M Wang H Hou S Hou G. Effects of *Lactobacillus delbrueckii* on performance, carcass traits and meat quality of ningxiang pigs. Chin J Anim Nutr. (2017) 29:325–32. 10.3969/j.issn.1006-267x.2017.12.038

[22] NRC. Nutrient Requirements of swine, 11th Edn.Washington, DC: National Academy Press (2012).

[23] Li R Chang L Hou G Song Z Fan Z He X. Colonic microbiota and metabolites response to different dietary protein sources in a piglet model. Front Nutr. (2019) 6:151. 10.3389/fnut.2019.00151 31616670 PMC6768948

[24] Li R Hou G Song Z Wu C Zhao J Sun X. Effects of different protein sources completely replacing fish meal in low-protein diet on growth performance, intestinal digestive physiology, and nitrogen digestion and metabolism in nursery pigs. Anim Sci J. (2019) 90:977–89. 10.1111/asj.13243 31199032

[25] Companys J Pla-Paga L Calderon-Perez L Llaurado E Sola R Pedret A. Fermented dairy products, probiotic supplementation, and cardiometabolic diseases: a systematic review and meta-analysis. Adv Nutr. (2020) 11:834–63. 10.1093/advances/nmaa030 32277831 PMC7360468

[26] Alves-Bezerra M Cohen DE. Triglyceride metabolism in the liver. Compr Physiol. (2018) 8:1–22. 10.1002/cphy.c170012 29357123 PMC6376873

[27] Hu Y Chen D Yu B Yan H Zheng P Mao X. Effects of dietary fibres on gut microbial metabolites and liver lipid metabolism in growing pigs. J Anim Physiol Anim Nutr (Berl). (2020) 104:1484–93. 10.1111/jpn.13429 32741066

[28] Munzberg H Singh P Heymsfield SB Yu S Morrison CD. Recent advances in understanding the role of leptin in energy homeostasis. F1000Res. (2020) 9:F1000 Faculty Rev-451. 10.12688/f1000research.24260.1 32518627 PMC7255681

[29] Kimura I Ozawa K Inoue D Imamura T Kimura K Maeda T. The gut microbiota suppresses insulin-mediated fat accumulation via the short-chain fatty acid receptor Gpr43. Nat Commun. (2013) 4:1829. 10.1038/ncomms2852 23652017 PMC3674247

[30] Shang P Wei MB Duan MQ Yan FF Chamba Y. Healthy gut microbiome composition enhances disease resistance and fat deposition in tibetan pigs. Front Microbiol. (2022) 13:965292. 10.3389/fmicb.2022.965292 35928149 PMC9343729

[31] Holman DB Brunelle BW Trachsel J Allen HK. Meta-analysis to define a core microbiota in the swine gut. mSystems. (2017) 2:e00004-17. 10.1128/mSystems.00004-17 28567446 PMC5443231

[32] Wang H Xu R Zhang H Su Y Zhu W. Swine gut microbiota and its interaction with host nutrient metabolism. Anim Nutr. (2020) 6:410–20. 10.1016/j.aninu.2020.10.002 33364457 PMC7750828

[33] Magne F Gotteland M Gauthier L Zazueta A Pesoa S Navarrete P. The Firmicutes/Bacteroidetes ratio: a relevant marker of gut dysbiosis in obese patients? Nutrients. (2020) 12:1474. 10.3390/nu12051474 32438689 PMC7285218

[34] Everard A Belzer C Geurts L Ouwerkerk JP Druart C Bindels LB. Cross-Talk between akkermansia muciniphila and intestinal epithelium controls diet-induced obesity. Proc Natl Acad Sci USA. (2013) 110:9066–71. 10.1073/pnas.1219451110 23671105 PMC3670398

[35] Wang K Liao MF Zhou N Bao L Ma K Zheng ZY. Parabacteroides distasonis alleviates obesity and metabolic dysfunctions *via* production of succinate and secondary bile acids. Cell Rep.(2019) 26:222. 10.1016/j.celrep.2018.12.028 30605678

[36] Hou G Li R Liu M Wang H Peng W Huang X. Effect of *Lactobacillus delbrueckii* on growth performance, nutrient digestibility, serun biochemical indexes and intestinal structure of fattening pigs. Chin J Anim Nutr. (2015) 27:2871–7. 10.3969/j.issn.1006-267x.2015.09.026

[37] Allen JM Berg Miller ME Pence BD Whitlock K Nehra V Gaskins HR. Voluntary and forced exercise differentially alters the gut microbiome in C57bl/6j mice. J Appl Physiol (1985). (2015) 118:1059–66. 10.1152/japplphysiol.01077.2014 25678701

[38] Koike S Ueno M Miura H Saegusa A Inouchi K Inabu Y. Rumen microbiota and its relation to fermentation in lactose-fed calves. J Dairy Sci. (2021) 104:10744–52. 10.3168/jds.2021-20225 34218911

[39] Liu H Wang J He T Becker S Zhang G Li D. Butyrate: a double-edged sword for health? Adv Nutr. (2018) 9:21–9. 10.1093/advances/nmx009 29438462 PMC6333934

[40] Schoeler M Caesar R. Dietary lipids, gut microbiota and lipid metabolism. Rev Endocr Metab Disord. (2019) 20:461–72. 10.1007/s11154-019-09512-0 31707624 PMC6938793

[41] Haenen D Zhang J Souza da Silva C Bosch G van der Meer IM van Arkel J. A diet high in resistant starch modulates microbiota composition, scfa concentrations, and gene expression in pig intestine. J Nutr. (2013) 143:274–83. 10.3945/jn.112.169672 23325922

[42] Li R Hou G Jiang X Song Z Fan Z Hou DX. Different dietary protein sources in low protein diets regulate colonic microbiota and barrier function in a piglet model. Food Funct. (2019) 10:6417–28. 10.1039/C9FO01154D 31517363

[43] Qian Y Li M Wang W Wang H Zhang Y Hu Q. Effects of lactobacillus casei ybj02 on lipid metabolism in hyperlipidemic mice. J Food Sci. (2019) 84:3793–803. 10.1111/1750-3841.14787 31762034

[44] Murphy KG Bloom SR. Gut hormones and the regulation of energy homeostasis. Nature. (2006) 444:854–9. 10.1038/nature05484 17167473

[45] Jiao AR Yu B He J Yu J Zheng P Luo YH. Short chain fatty acids could prevent fat deposition in pigs via regulating related hormones and genes. Food Funct. (2020) 11:1845–55. 10.1039/C9FO02585E 32067021

[46] Zechner R Zimmermann R Eichmann TO Kohlwein SD Haemmerle G Lass A. Fat signals - lipases and lipolysis in lipid metabolism and signaling. Cell Metab. (2012) 15:279–91. 10.1016/j.cmet.2011.12.018 22405066 PMC3314979

[47] Aronsson L Huang Y Parini P Korach-Andre M Hakansson J Gustafsson JA. Decreased fat storage by lactobacillus paracasei is associated with increased levels of angiopoietin-like 4 protein (Angptl4). Plos ONE. (2010) 5:e13087. 10.1371/journal.pone.0013087 20927337 PMC2948012

[48] Hardie DG Schaffer BE Brunet A. Ampk: an energy-sensing pathway with multiple inputs and outputs. Trends Cell Biol. (2016) 26:190–201. 10.1016/j.tcb.2015.10.013 26616193 PMC5881568

[49] Hao JW Wang J Guo H Zhao YY Sun HH Li YF. CD36 facilitates fatty acid uptake by dynamic palmitoylation-regulated endocytosis. Nat Commun. (2020) 11:4765. 10.1038/s41467-020-18565-8 32958780 PMC7505845

[50] Cui JX Zeng QF Chen W Zhang H Zeng YQ. Analysis and preliminary validation of the molecular mechanism of fat deposition in fatty and lean pigs by high-throughput sequencing. Mamm Genome. (2019) 30:71–80. 10.1007/s00335-019-09795-3 30843090 PMC6491413

