# Supplementary material for: Lactobacillus delbrueckii might lower serum triglyceride levels via colonic microbiota modulation and SCFA-mediated fat metabolism in parenteral tissues of growing-finishing pigs
Source: Front Vet Sci. 2022 Sep 29;9:982349. doi: 10.3389/fvets.2022.982349 (PMC9557183; doi:10.3389/fvets.2022.982349)
Supplement: Supplementary file 1 [file Data_Sheet_1.docx]

**SUPPLEMENTARY TABLE 1 |** Effects of *Lactobacillus delbrueckii* on growth performance of growing-finishing pigs

| Items | Con | Con+LD | P-Value |
| --- | --- | --- | --- |
| Initial body weight (IBW, kg) | 38.90±6.32 | 38.50±4.90 | 0.638 |
| Final body weight (FBW, kg) | 68.60±9.22 | 71.60±3.42 | 0.158 |
| Average daily gain (ADG, g) | 1060.71±183.24 | 1182.14±70.76 | 0.239 |
| Average daily feed intake (ADFI, g) | 2671.43±302.41 | 2889.29±123.96 | 0.248 |
| Feed to gain ratio (F/G) | 2.55±0.23 | 2.45±0.22 | 0.741 |

Con = basal diet; Con + LD = basal diet + 0.1 % *Lactobacillus delbrueckii.*

**SUPPLEMENTARY TABLE 2 |** Relative abundance of phylum and genus level in colonic digesta.

| Items | Con | Con+LD | P-Value |
| --- | --- | --- | --- |
| Phylum level | | | |
| Firmicutes | 0.7169±0.1288 | 0.5393±0.0307 | 0.092 |
| Verrucomicrobia | 0.0814±0.1030 | 0.1874±0.0141 | 0.060 |
| Bacteroidetes | 0.1426±0.0446 | 0.2065±0.0326 | 0.868 |
| Proteobacteria | 0.0384±0.0145 | 0.0311±0.0215 | 0.551 |
| Euryarchaeota | 0.0041±0.0036 | 0.0147±0.0147 | 0.001 |
| Spirochaetes | 0.0052±0.0015 | 0.0107±0.0083 | <0.001 |
| Actinobacteria | 0.0053±0.0015 | 0.0063±0.0045 | 0.112 |
| Tenericutes | 0.0043±0.0015 | 0.0018±0.0006 | 0.278 |
| unidentified_Bacteria | 0.0008±0.0004 | 0.0007±0.0004 | 0.694 |
| Melainabacteria | 0.0002±0.0001 | 0.0001 | 0.096 |
| Genus level | | | |
| *Akkermansia* | 0.0814±0.1030 | 0.1874±0.0141 | 0.060 |
| *Romboutsia* | 0.1054±0.0890 | 0.0464±0.0092 | 0.049 |
| *unidentified_Clostridiales* | 0.1872±0.0483 | 0.1130±0.0179 | 0.049 |
| *Streptococcus* | 0.0053±0.0083 | 0.0467±0.0300 | 0.005 |
| *unidentified_Ruminococcaceae* | 0.0920±0.1627 | 0.0010±0.0003 | 0.065 |
| *unidentified_Prevotellaceae* | 0.0079±0.0082 | 0.0034±0.0270 | 0.121 |
| *Lactobacillus* | 0.0193±0.0099 | 0.0349±0.0238 | 0.005 |
| *Bacteroides* | 0.0158±0.0127 | 0.0270±0.0179 | 0.735 |
| *unidentified_Lachnospiraceae* | 0.0198±0.0137 | 0.0280±0.0193 | 0.352 |
| *Blautia* | 0.0225±0.0128 | 0.0244±0.0066 | 0.212 |
| *Parabacteroides* | 0.0046±0.0045 | 0.0171±0.0174 | 0.099 |
| *Turicibacter* | 0.0253±0.0075 | 0.0181±0.0038 | 0.074 |
| *unidentified_Enterobacteriaceae* | 0.0209±0.0091 | 0.0171±0.0130 | 0.339 |
| *Megasphaera* | 0.0012±0.0026 | 0.0107±0.0120 | 0.077 |
| *unidentified_Erysipelotrichaceae* | 0.0003±0.0004 | 0.0071±0.0139 | 0.033 |
| *Methanobrevibacter* | 0.0041±0.0036 | 0.0147±0.0147 | 0.001 |
| *Terrisporobacter* | 0.0074±0.0019 | 0.0092±0.0076 | 0.129 |
| *unidentified_Spirochaetaceae* | 0.0029±0.0011 | 0.0058±0.0052 | 0.003 |
| *Alloprevotella* | 0.0054±0.0039 | 0.0053±0.0020 | 0.240 |
| *Butyrivibrio* | 0.0005±0.0001 | 0.0033±0.0035 | 0.016 |

Con = basal diet; Con + LD = basal diet + 0.1 % *Lactobacillus delbrueckii*.

**SUPPLEMENTARY TABLE 3 |** Effects of *Lactobacillus delbrueckii* on TG concentrations in different tissue of growing-finishing pigs (mmol/g.prot).

| Items | Con | Con+LD | P-Value |
| --- | --- | --- | --- |
| Subcutaneous fat | 1.49±0.52 | 1.93±0.74 | 0.134 |
| *Longissimus dorsi* | 0.50±0.10 | 0.56±0.08 | 0.295 |
| Leaf lard | 1.34±0.54 | 1.32±0.29 | 0.119 |
| Liver | 0.44±0.07 | 0.52±0.10 | 0.100 |
